# Disentangling Capabilities for Industry 4.0 - an Information Systems Capability Perspective

**DOI:** 10.1007/s10796-022-10260-x

**Published:** 2022-04-02

**Authors:** Rocco Huber, Anna Maria Oberländer, Ulrich Faisst, Maximilian Röglinger

**Affiliations:** 1 FIM Research Center, University of Augsburg, Project Group Business & Information Systems Engineering of the Fraunhofer FIT, Augsburg, Germany; 2 FIM Research Center, University of Bayreuth, Project Group Business & Information Systems Engineering of the Fraunhofer FIT, Wittelsbacherring 10, 95444 Bayreuth, Germany; 3 Chief Technology Officer, Cognizant Technology Solutions GmbH, Frankfurt, Germany

**Keywords:** Industry 4.0, Fourth industrial revolution, Information systems capabilities, Capability framework

## Abstract

Digital technologies revolutionise the manufacturing industry by connecting the physical and digital worlds. The resulting paradigm shift, referred to as Industry 4.0, impacts manufacturing processes and business models. While the ‘why’ and ‘what’ of Industry 4.0 have been extensively researched, the ‘how’ remains poorly understood. Manufacturers struggle with exploiting Industry 4.0’s full potential as a holistic understanding of required Information Systems (IS) capabilities is missing. To foster such understanding, we present a holistic IS capability framework for Industry 4.0, including primary and support capabilities. After developing the framework based on a structured literature review, we refined and evaluated it with ten Industry 4.0 experts from research and practice. We demonstrated its use with a German machinery manufacturer. In sum, we contribute to understanding and analysing IS capabilities for Industry 4.0. Our work serves as a foundation for further theorising on Industry 4.0 and for deriving theory-led design recommendations for manufacturers.

## Introduction

Technological leaps have always had the power to affect entire industries implying paradigm shifts (Lasi et al., 2014; Zhong et al., 2017). Indeed, leaps in technology have led to industrial revolutions with enormous ramifications for production methods, value chains, and social structures (Baines et al., 2017; Kazancoglu & Ozkan-Ozen, 2018). Within the last decade, digital technologies such as cloud computing, the Internet of Things (IoT), and artificial intelligence (AI) have brought our physical and digital worlds into close contact, so much so that they have triggered the fourth industrial revolution – also known as Industry 4.0 (Ghobakhloo, 2018; Weking et al., 2020). Products are now increasingly complemented with digital services (Culot et al., 2019; Zhong et al., 2017), which changes how manufacturers invent, create, and deliver products and services (Lasi et al., 2014; Xu et al., 2018). Academics and practitioners alike expect Industry 4.0 to transform the manufacturing industry in fundamental ways that extend as far as to its revenue and efficiency potential (Weking et al., 2020; Zhang et al., 2021). Exemplary use cases include machine monitoring (Momeni & Martinsuo, 2018), predictive maintenance (Baptista et al., 2018), and smart process planning (Bellini et al., 2021; Trstenjak & Cosic, 2017).

Despite its enormous potential, only a minority of manufacturers have made a successful transformation with the full benefits of Industry 4.0 technologies, applications, and digital product solutions (Moeuf et al., 2020). This transformation rate is especially low among small- and medium-sized manufacturers. It is worth noting that realising the potential of Industry 4.0 requires adaptation on the part of manufacturers as they have to complement their strengths in manufacturing-related core capabilities with new Information Systems (IS) capabilities (Baines et al., 2017; Bustinza et al., 2017). Significant challenges associated with this stem from a lack of awareness and understanding of the IS capabilities involved in the Industry 4.0 transformation (Ghobakhloo, 2018; Lund & Karlsen, 2020). It follows that for manufacturers to make good, far-sighted decisions regarding organisational, management, and employee development, they would be well served with a holistic capability overview. Rather than focusing more on the well-known ‘why’ and ‘what’ of Industry 4.0, this study addresses the neglected ‘how’. The study aims to provide a holistic perspective on the IS capabilities required by manufacturers that wish to make the most of Industry 4.0 (Moeuf et al., 2020).

As far as the theory goes, the literature on Industry 4.0 can be deemed mature since it provides relevant knowledge on how to describe and structure various elements of Industry 4.0 (Alcácer & Cruz-Machado, 2019; Oztemel & Gursev, 2020). For example, researchers have outlined multiple success factors (Shinohara et al., 2017) and requisite employee qualifications (Lund & Karlsen, 2020; Prifti et al., 2017). The existing body of knowledge also covers important transformational aspects, be it in the form of an Industry 4.0 readiness assessment (Wagire et al., 2020) or overarching maturity models (Schuh et al., 2020; Schumacher et al., 2016). Although these studies provide a good understanding of Industry 4.0 transformations, they do not cover all – or even all that many – of the IS capabilities required to run a successful business in the age of Industry 4.0. The purpose of this paper is to offer a theoretically well-founded overview of IS capabilities, not only to support manufacturers in practice but also to set out a foundation on which others can further advance the theory on Industry 4.0, be it by explaining related success factors, predicting outcomes, or deriving recommendations for design and action (Gregor, 2006). The research challenge is to identify, structure, and better understand those IS capabilities. With that in mind, our research question is as follows: *Which IS capabilities do manufacturers need to realise Industry 4.0?*

To answer this question, we iteratively developed and evaluated a conceptual framework for the IS capabilities that manufacturers need to make the most of Industry 4.0. The resulting IS capability framework for Industry 4.0 is strengthened by thorough theoretical research, having first been developed in line with a structured literature review that took special note of Webster and Watson (2002) and Wolfswinkel et al. (2013). To further evaluate the framework on its relevance, clarity, and complementary capabilities that had yet to be considered in the literature, we conducted 10 semi-structured interviews with a range of experts at work in academia and industry. As for the framework’s practical use, we demonstrate a possible application of the framework with a German manufacturer of metal and tube processing solutions.

To summarise, our work contributes a theoretically well-founded and practically relevant IS capability framework for Industry 4.0. Although there is substantial work and knowledge on Industry 4.0 (e.g., Duan & Xu, 2021; Li, 2018; Weking et al., 2020; Xu et al., 2018), the framework is novel as it provides a range of requisite capabilities by taking an overdue IS perspective on Industry 4.0. Second, by evaluating and iteratively developing this framework in consultation with a diverse panel of senior industry executives, we were able to complement capabilities neglected by research so far and also ensure a high practical value of the framework. Third, its modular nature makes it a suitable foundation for further theorising on Industry 4.0, which will aid anyone wishing to understand, for instance, the dependencies of capabilities and derive design actions for manufacturers to guide their Industry 4.0 transformations. Fourth, this framework supports practitioners not only in recognising and comprehending all of the relevant IS capabilities for their Industry 4.0 transformations. It also supports them in assessing and developing the necessary IS capabilities and guides them through the vagaries of transforming their organisation.

The remainder of this paper is structured as follows: In Section 2, we elaborate on the theoretical background of Industry 4.0, IS capabilities, and capability frameworks. In Section 3, we outline our research method. In Sections 4 and 5, we present and discuss the IS capability framework for Industry 4.0, including managerial and theoretical implications, after which we conclude by stating our study’s limitations and providing pointers for future research.

## Theoretical Background

### Industry 4.0

Industry 4.0 is driven by the rapid emergence and adoption of digital technologies in the industrial sector where it is used to foster (industrial) information integration (Javaid et al., 2021) and the digitalisation of manufacturing (Li, 2018; Vaidya et al., 2018). Its technical core foundation is the IoT, which facilitates connections, communication, and control among physical objects, people, systems, and IT (Oberländer et al., 2018; Xu et al., 2018). In the context of Industry 4.0, the IoT is often referred to as ‘industrial IoT’ (IIoT) or cyber-physical systems (CPS) (Duan & Xu, 2021; Xu et al., 2018). IIoT and CPS are understood as technological systems that consist of physical parts, embedded sensors and actuators, and computing logic. They are instruments of integrating virtual space into the physical world (Berger et al., 2021; Duan & Xu, 2021). Rather than simply being off-the-shelf technologies, they rely on integrating machine-to-machine communication, industrial controllers, cloud data, big data analytics, and semantic technologies (Zhang et al., 2021). Working in conjunction with another, they create a dynamic cyber-physical control system (Lu, 2017a, 2017b) that opens up new ways of producing and using physical products (Berger et al., 2021; Zhang et al., 2021). Exemplary use cases include data-based monitoring, planning and decision-making processes, and predictive maintenance services for products. Whereas terms like IIoT and CPS are clearly defined and often used synonymously (Menon et al., 2020), Industry 4.0 has yet to be determined beyond ambiguity (Ghobakhloo, 2018; Maresova et al., 2018).

To date, the expert literature has recorded three notable definitions of Industry 4.0: (1) the process of digitalising the manufacturing industry (Cohen et al., 2019; Oesterreich & Teuteberg, 2016); (2) a new paradigm for industrial production with a focus on the process outcome (Kagermann, 2015; Vaidya et al., 2018); (3) a combination of those two perspectives (i.e. transformation process and its outcome). In accordance with this third definition, Industry 4.0 is used as an umbrella term for new technologies and concepts in manufacturing (Hermann et al., 2016). As we understand it, the term Industry 4.0 covers both digital transformation (process perspective) and a new manufacturing paradigm (outcome perspective). All three definitions agree that Industry 4.0 has three central characteristics: the horizontal integration of manufacturing and service systems, their vertical integration, and the end-to-end digitalisation of processes (Zhang et al., 2021). Horizontal integration occurs mainly on the production floor and across multiple production facilities, where it connects manufacturing processes based on digital technologies and shared data. On the other hand, vertical integration occurs between numerous layers within one company (e.g. production, finance) and across its value chain to connect production and service systems. The final characteristic – end-to-end digitalisation of the complete value chain – is about digitalising processes and products to enable scalable manufacturing and service systems (Stock & Seliger, 2016; Weking et al., 2020). With this in mind, we understand Industry 4.0 as the *digitalisation of products, production facilities, and value chains, made possible by the connection of people, objects, and systems, which in turn is made possible by the extensive integration of digital technologies* (Hermann et al., 2016; Lasi et al., 2014; Ransbotham et al., 2016; Xu et al., 2018). In other words, we understand it as the next stage in rethinking and controlling a manufacturing company’s entire value network, including customers and business partners. To be clear, we include the strategic dimension and company ecosystem but exclude societal and ethical aspects. The latter constitutes the main difference to the digital transformation concept, which also builds on digital technologies to enhance existing capabilities or create new ones (Sebastian et al., 2017). As opposed to our framework, however, this concept involves ‘broader individual, organisational, societal contexts’ (Legner et al., 2017, p. 301) and has a rather company-centric perspective (Vial, 2019).

### Information Systems Capabilities

Recently, management literature has highlighted the importance of mastering the many ways in which digital technologies can respond to changing environmental conditions (Khin & Ho, 2019; Kohli & Melville, 2019). There is an interesting parallel between industries such as telecommunications, media, and financial services, where digital technologies give technophile competitors a lucrative advantage over the competition. In this context, the resource-based view (Wernerfelt, 1984) has been widely used to explain superior performance in the market by shifting the focus to the effective use of digital technologies (Khin & Ho, 2019; Levallet & Chan, 2018). The theory is that a company’s performance can be attributed to the effective use of any assets and capabilities that are company-specific, rare, and difficult to imitate by others (Barney, 1991). Whereas assets can be tangible as well as intangible, capabilities are ‘repeatable patterns of actions in the use of assets to create, produce, and/or offer products to a market’ (Wade & Hulland, 2004, p. 209). Alternatively, capabilities refer to the different ways in which a company can perform a coordinated set of tasks to achieve a particular result (Helfat & Peteraf, 2003). With specific regard to digital technologies, capabilities can be defined as ‘the ability to mobilise and deploy IT-based resources in combination or co-present with other resources and capabilities’ (Bharadwaj, 2000, p. 171). For the most part, we agree with this definition, yet we expand it in a crucial sense to include the ability to facilitate business opportunities by making the most of digital technologies. For the remainder of this paper, we will refer to IS capability as a *company’s ability to assemble, integrate, deploy, and connect digital technologies, thereby enabling the realisation of the company’s Industry 4.0 strategy to enhance new business opportunities* (Bharadwaj, 2000; Peppard & Ward, 2004; Sambamurthy et al., 2003).

Capability frameworks are commonly used to structure and describe capabilities, then group them according to similarity into larger capability areas (Hosseini et al., 2017; Rosemann & vom Brocke, 2015). These frameworks consist of structuring elements that differ in granularity. For this study, we use Porter’s (1998) value chain to structure all IS capabilities relevant to Industry 4.0. Porter’s (industry) value chain reflects a set of activities and processes that a company operates in a specific manner to produce valuable products, services, or any combination of the two. Activities are classified into primary activities (e.g. operations and sales) and support activities (e.g. technology and human resources) (Porter, 1998). Primary activities can add value and create a competitive advantage, whereas support activities can increase the effectiveness of primary activities. This general perspective, widely applied to such purposes as strategy assessments, draws out the interconnections between customers, suppliers, and other value-creating partners. It also makes it easier to structure the framework in a comprehensive manner and apply it in practice.

### Capability Frameworks for Industry 4.0

To avoid reinventing the wheel in this paper, it is worth pointing out that the literature on Industry 4.0 has reached a maturity level at which it already provides sufficient information on the ‘what’ (e.g. digital technologies) and the ‘why’ (e.g. success factors) of the Industry 4.0 transformation of manufacturers (Alcácer & Cruz-Machado, 2019; Oztemel & Gursev, 2020). For example, Shinohara et al. (2017) outlined critical success factors for digital manufacturing, such as IT infrastructure, training programs, and the effective appointment of project teams. However, what has yet to be worked out in adequate detail is the ‘how’ of Industry 4.0, which is to say, the broad range of underlying capabilities required to realise the identified success factors.

In the context of Industry 4.0 capabilities, most current publications fall into the category of conceptual work (i.e., frameworks and maturity models). While some identify and describe several required capabilities, they do so mainly from a technical, anecdotal, and practitioner-oriented perspective. For example, Stich et al. (2017) constructed a capability framework that was to be used for information management to achieve maturity in Industry 4.0, yet their view of this broad matter rather favoured the narrow field of technical aspects. Similarly, Prifti et al. (2017) developed a competency model for Industry 4.0 and structured it into the three major domains of IS, Computer Science, and Engineering, and rather than looking at the bigger picture, they limited the scope of their study to employee competencies and how these must be further developed and combined. Meanwhile, Schumacher et al. (2016) broadened the prevalent focus on technology. Looking beyond the fundamental enablers ‘Products’, ‘Customers’, ‘Operations’ and ‘Technology’, the assessment of ‘Strategy’, ‘Leadership’, ‘Governance’, ‘Culture’ and ‘People’ involves organisational aspects. However, these relevant capabilities are only mentioned in an anecdotal manner rather than described in more detail. Similarly, the application-focused maturity model of the German National Academy of Science, developed by (Schuh et al., 2020), takes a rather practitioner-oriented perspective, dedicating most of its attention to potential implementation pathways or exemplary technologies that may be of use in the area of application. Another disadvantage worth observing here is that the narrow foundation laid in this work is not embedded in the (academic) literature, making it difficult for further research to build upon. Meanwhile, Wagire et al. (2020) devised a maturity model to assess the Industry 4.0 progress of manufacturers by developing 38 maturity items grouped into seven dimensions. Although they concentrated on technology-focused capabilities, they also included aspects such as ‘people and culture’ and ‘organisational strategy’. Unfortunately, they assessed Industry 4.0 maturity without providing a well-founded and theory-grounded overview of the capabilities on which such a maturity model is predicated.

Based on this assessment of related literature, we conclude that the work that has so far been done on Industry 4.0 capabilities has provided some useful initial insights into how one can make the most of Industry 4.0 from a technical, anecdotal, and practitioner-oriented perspective. What appears to be overdue, however, is a comprehensive and theoretically well-founded account of the IS capabilities required to realise Industry 4.0. An account from which both researchers and practitioners can extrapolate methods to improve and optimise business structures in this new industrial era.

## Research Method

**Fig. 1:**
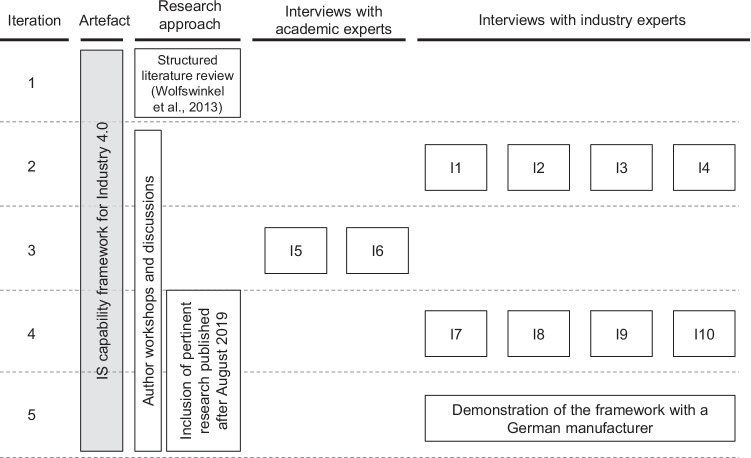
Framework development process

Because the emerging field of IS capabilities in Industry 4.0 is interdisciplinary in nature, we set out to develop and evaluate the ideal framework in consultation with researchers as well as practitioners. It soon became apparent that this would require a multi-step research process (Fig. 1), so we first performed a structured literature review in line with Webster and Watson (2002) and Wolfswinkel et al. (2013), the purpose being to identify the IS capabilities relevant to Industry 4.0. With the benefit of those insights, we coded and structured significant findings to draft an initial version of the framework. To then evaluate this framework in the course of several iterations, we conducted ten semi-structured interviews, eight of which involved industry experts from manufacturing companies, while the remaining two drew on the wide-ranging experience of senior scholars from the IS domain. All insights from these interviews and the improvements were discussed in detail by the author team and substantiated with regard to the domain literature. In the final analysis, we accounted that Industry 4.0 is a fast-developing topic, so we went back to screen expert literature once more and included any relevant to our research question.

**Fig. 2:**
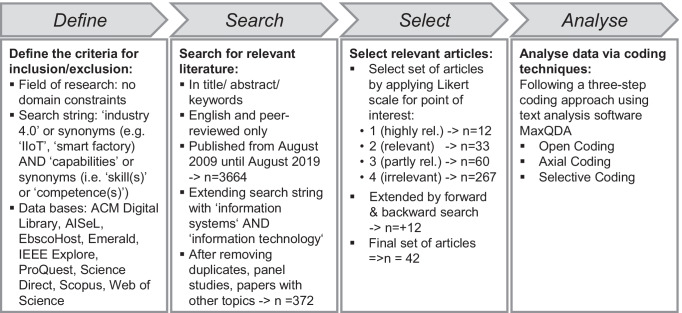
Structured literature review approach based on Wolfswinkel et al. (2013)

For the initial version of the framework, we went through the relevant literature to conduct a thorough analysis based on Wolfswinkel et al. (2013). This process consists of five stages: define, search, select, analyse, and present. In Fig. 2, we briefly describe what occurred at each of these stages, except the final one – ‘present’ – since this entire paper is written to deal with that, i.e., the presentation and communication of results to one’s target audience.

At the first stage, ‘**Define**’, the relevant field of research has to be found. Because our topic holds great relevance in a broad range of domains, such as IS, engineering, and economics, we approached it from an interdisciplinary angle. We searched for articles in nine different research databases, with no specific restrictions on journals and conferences (see Fig. 2). We decided to include conference proceedings as well to ensure we would account for the most recent developments. To process this wealth of data, we carefully selected appropriate search terms and refined them through various iterations (Wolfswinkel et al., 2013). The final search string included ‘Industry 4.0’, synonyms such as ‘smart factory’ or ‘industrial Internet of Things’. It also included the term ‘capabilities’ along with the synonyms ‘competence(s)’ or ‘skill(s)’.

At the second stage, ‘**Search**’, we applied our search string to the selected databases, which yielded an initial sample of 56,614 articles. To narrow this down to the essential literature that was rightly qualified for consideration in this study, we selected the material that included our search terms in the title, abstract, or keywords (Wolfswinkel et al., 2013). In a further measure to refine the literature’s relevance and to ensure that researchers who wish to carry on our work in this highly topical area can read this study and find therein a complete account of the most current thoughts on the key issues, we limited our search of the literature to the past ten years, i.e., from August 2009 to August 2019. We also added the search terms ‘information systems’ and ‘information technology’ to sharpen the focus on IS capabilities.

At the third stage, ‘**Select**’, we carefully reviewed all titles and abstracts to identify relevant articles and evaluate their relevance (Wolfswinkel et al., 2013). We operationalised this approach by using a four-point Likert scale, the score ranging from 1 to 4. An article scored a rating of 4 if the abstract addressed IS capabilities in the context of Industry 4.0, whereas if there was no connection to the research question, an article would score a rating of 1. Due to this selection process, we identified 45 articles (score 2 and 1) worthy of in-depth screening, 30 of which we ultimately deemed relevant to this study. Finally, we conducted a forward and backward search, in the course of which we found 12 further publications that warranted inclusion in our research.

At the fourth stage, ‘**Analyse**’, we followed Wolfswinkel et al. (2013), proposing a three-step coding approach. We used open, axial, and selective coding to analyse our literature sample. Starting with open coding, we read each publication with close attention to detail and highlighted any findings relevant to our research question. Continuing with axial coding, we identified the main categories and their relations, whereupon we completed the coding process by deriving the key IS capabilities from the main types, refining them through various iterations, and selecting those upon which the author team could achieve a consensus. By cross-referencing our thoughts with established theories from (non-)IS domains, we finally concluded that the value chain of Porter (1998) best structures the relevant IS capabilities, seeing as it considers both primary and support capabilities. With this in mind, we carefully assessed each capability and consolidated them in the author team.

**Fig. 3:**
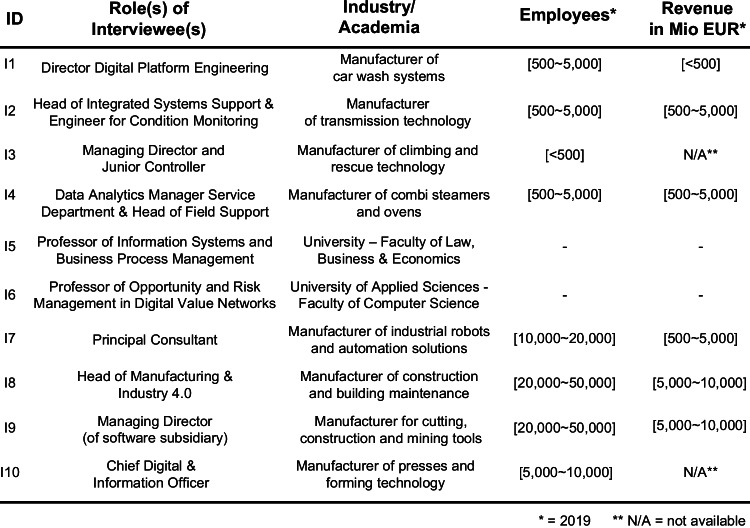
Overview of interviews with industry and academic experts

To subject this theoretically developed framework to the hard test of practical experience and critical evaluation, we conducted ten semi-structured interviews (Myers & Newman, 2007), as shown in Fig. 3. These interviews also revealed further capabilities which had to be included in the scope of this study yet which, due to the topic’s relative novelty and rapid development, had not yet been addressed to a sufficient extent by the larger research community. These interviews were conducted by a minimum of two researchers and lasted between 1 and 1.5 h, either in face-to-face conversations or via video conferences. In each interview, we presented the framework and discussed all of the capabilities in terms of problem relevance to the problem and their understanding (Cole et al., 2005; March & Smith, 1995; Peffers et al., 2007). We also inquired about IS capabilities that may have been missing from the framework and asked the interviewees to challenge the design of the framework. After each interview, the author team discussed any new insights, searched for confirmation in the expert literature, and proposed changes to the framework accordingly. In addition, to test whether the framework is understandable and applicable (Sonnenberg & vom Brocke, 2012), it was applied with the CTO of a German manufacturer where a thorough capability assessment was carried out. The objective was to identify the status quo of a company’s relevant IS capabilities, define its desired target states in 3 years’ time, and reflect on the associated chances and challenges. Once we no longer received new insights from the interviewed experts nor saw any other occasion for significant changes to the framework, we reviewed all recently published work by searching Google Scholar with regard to each capability. We filtered our findings by focusing on work published within the last 4 years and then selecting only the highly referenced studies (i.e. more than ten citations).

## Information Systems Capability Framework for Industry 4.0

**Fig. 4:**
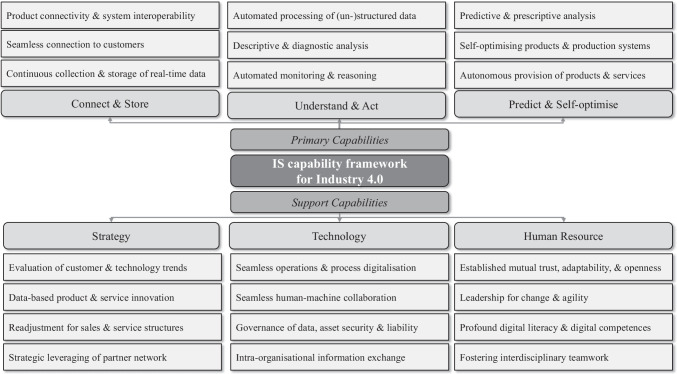
IS capability framework for Industry 4.0

In the following, we present the IS capability framework for Industry 4.0 (Fig. 4). To provide a holistic perspective on the company and its role within its value network, including interconnections with customers and other partners, we decided to structure the IS capabilities for Industry 4.0 following Porter’s (1998) value chain model. In doing so, we distinguish between primary and support capabilities. Primary capabilities focus on the supply of new product and service solutions as well as the technical challenges of putting them into practice. In contrast, support capabilities focus on improving support processes and collaboration in and beyond the company to increase the effectiveness of primary capabilities. The industry experts consulted for this study highlighted the importance of support capabilities and identified their disregard in existing (practical) approaches as one of the main reasons for the relatively slow progress in Industry 4.0.

In dealing with primary capabilities, we agree with Porter’s (1998) understanding of primary activities to the extent that they are essential to adding value and creating competitive advantage. Porter (1998) differentiates primary activities into a chronological sequence, from inbound logistics to after-sales services for (physical) products. However, since the activities involved in manufacturing physical products bear no direct comparison with the creation of digital offerings, we adapted this classification of activities (i.e. capabilities) by drawing on the work of Porter and Heppelmann (2015) and Schuh et al. (2020). Both describe and structure analytical (Porter & Heppelmann, 2015) and IS capabilities (Schuh et al., 2020) that can be used to complement physical products with digital services. With this in mind, we differentiate the primary capabilities as follows:

- **Connect & Store:** Fundamental capabilities required to manage the technology stack for assessing and storing data (Schuh et al., 2020).
- **Understand & Act:** Basic capabilities used to automatically process data or serve descriptive and diagnostic purposes (Porter & Heppelmann, 2015; Schuh et al., 2020).
- **Predict & self-optimise:** Extended analytical capabilities that serve predictive and prescriptive purposes or enable autonomous systems (Porter & Heppelmann, 2015; Schuh et al., 2020).

Whereas primary capabilities are essential for adding value and creating a competitive advantage, support capabilities increase the effectiveness of primary capabilities. To take account of this, we adapted the theory that Porter (1998) proposed as a way to transform and optimise various company’s functions, such as finance, controlling, and marketing. Instead, we reconfigured this as follows:

- **Strategy:** Capabilities related to general (strategic) management and help to define and manage the Industry 4.0 transformation.
- **Technology:** Capabilities related to technical knowledge and help digitalise support processes and ensure the safe use of Industry 4.0 technologies.
- **Human Resource:** Capabilities related to cultural aspects, including the understanding, acceptance, and use of technology.

Tables 1 and 2 define each capability, then provide short explanations and examples drawn from the literature and industry experts interviewed. We chose a high level of abstraction for the descriptions to ensure their applicability in various manufacturing industries and contexts. Further worth noting is that relationships might emerge among the IS capabilities or natural orders in which they might be implemented. With this in mind, we do not suggest a strict sequence in which these capabilities must be developed, nor that they necessarily depend on one another.

**Table 1:** Overview of primary IS capabilities for Industry 4.0

Primary IS capabilities for Industry 4.0		
Capability	Definition	Explanation & Example(s)
Connect & Store		
Product connectivity & system interoperability	A company’s ability to establish connectivity between systems, physical objects, and humans Also, the ability of products to exchange data via defined and standardised interfaces (Givehchi et al., 2017; Javaid et al., 2021; Schuh et al., 2020).	*Explanation:* Connectivity is one of Industry 4.0’s most essential building blocks. To enable the efficient sharing, exchange, and management of data (e.g. product lifecycle data), a high value must be placed on interoperability and communication standards (Givehchi et al., 2017; Schuh et al., 2020). Meanwhile, transparency and traceability can be increased by integrating sensors, actuators, and computing capacities into physical objects (Bienhaus & Haddud, 2018). *Example(s):* Product components store location, process, and manufacturing data (Man & Strandhagen, 2017). Implementation ranges from RFID technology and smart sensors (I1, I3) to machine-to-machine communication interfaces as integral product components (I4).
Seamless connection to customers	A company’s ability to remotely monitor product usage. Also, the ability to acquire and analyse data on customer needs (Beverungen et al., 2019; Siggelkow & Terwiesch, 2019)	*Explanation:* With digital technologies, connections to customers can be made continuous and seamless. They can even be reconfigured as individualised interactions, allowing companies to proactively address customer needs as they arise (Siggelkow & Terwiesch, 2019). To achieve this, data gathered through connected products can be used to understand better and predict customer needs (Beverungen et al., 2019). While this capability has not yet been explored at any great depth in the literature, it was emphasised by all of our interviewed industry experts. *Example(s):* Expert I1 drew our attention to a car wash installation that monitors various operating criteria 24/7, i.e., the car wash’s usage, customers, car visits, and required chemicals. This enables the manufacturer to adjust their services, such as seasonal wash programmes, to the demands of individual customers.
Continuous collection & storage of real-time data	A company’s ability to collect and store real-time data from manufacturing processes and connected products (Emmanouilidis et al., 2019; Javaid et al., 2021; Lasi et al., 2014; Schuh et al., 2020)	*Explanation*: Most Industry 4.0 services and benefits rely on product and process data, which is why the collection and storage of structured and unstructured data is a core capability (Alcácer & Cruz-Machado, 2019; Schuh et al., 2020). Due to new possibilities in exchanging real-time data (e.g. cloud solutions), the amount of stored data grows exponentially as single entities and data from entire manufacturing processes, plants, and connected products can be stored (Emmanouilidis et al., 2019; Lasi et al., 2014). *Example(s*) For example, the storage of traceable event timelines enables the verification of data evaluation and recovery of technical issues and evidence-based decision-making (I2).
Understand & Act		
Automated processing of (un-)structured data	A company’s ability to derive meaningful information from (un-)structured data from different sources (Alcácer & Cruz-Machado, 2019; Prifti et al., 2017; Schuh et al., 2020).	*Explanation:* The transformation of data into information, which can require the processing of large amounts of data, includes the categorisation, characterisation, consolidation, and classification of data. On the one hand, structured data (e.g. ERP data) allows for quick aggregation of data from different machines, products, and systems into a unified format that facilitates more straightforward analysis. Such automation is necessary to efficiently process the ever-increasing amount of input without human intervention (Prifti et al., 2017). On the other hand, mainly processing unstructured data is an even more significant challenge with enormous potential (Alcácer & Cruz-Machado, 2019). Our industry experts especially emphasised the opportunity in analysing unstructured data. *Example(s):* To automate processes, some of our interviewed experts suggested the use of data from (technical) service reports that contain both structured and unstructured data (I1, I4).
Descriptive & diagnostic analysis	A company’s ability to analyse product and process data in such a way as to easily detect (unwanted) deviations and identify their root causes (Dai et al., 2020; Duan & Xu, 2021; Porter & Heppelmann, 2014; Shi-Nash & Hardoon, 2017).	*Explanation:* Using statistical methods and descriptive analysis of historical data (e.g. product lifecycle data, manufacturing processes), manufacturers can better detect unwanted deviations from a predefined standard (Dai et al., 2020; Shi-Nash & Hardoon, 2017). This involves the description of KPIs (e.g. for a manufacturing process), visualisation of machine states, and detecting machinery failures as well as any anomalies or patterns in the data. Once a malfunction or failure is detected, the diagnostic analysis attempts to identify the root cause (Dai et al., 2020). The diagnostic analysis is a reactive response (Porter & Heppelmann, 2014), much like descriptive analysis and greatly depends on human-expert reasoning. *Example(s):* A manufacturer of transmission technology (I2) performs diagnostic services for customers by detecting anomalies in the vibration patterns of their transmission systems (gained from sensor data). However, an engineer is always required to analyse and further evaluate the data in order to identify the root cause.
Automated monitoring & reasoning:	A company’s ability to automatically monitor manufacturing processes and products to optimise their reasoning and act in a way that positively impacts production as well as decision-making processes (Babiceanu & Seker, 2016; Wagire et al., 2020; Waschull et al., 2020)	*Explanation:* Advanced and embedded analytics tools enable automatic monitoring of products and manufacturing processes, which in turn enables real-time decision-making in dealing with unwanted deviations (Waschull et al., 2020). Such systems aim to minimise human involvement by combining detection and reasoning. If a problem occurs for a specific entity, the monitoring system detects this critical event automatically. Based on predefined constraints and the identified reason, the system then triggers predefined processes (Babiceanu & Seker, 2016). *Example(s):* A car wash manufacturer uses the continuous condition monitoring of a car wash system in conjunction with data analytics to detect customer-specific sources of component failures (I1). This lays the foundation for novel services, such as the automated ordering of spare parts. This provides an ‘added value’ (I8) for the customer and higher profit margins for manufacturers.
Predict & Self-optimise		
Predictive & prescriptive analysis	A company’s ability to automatically predict events and time-series based on data-driven methods, then suggest optional decisions based on the results (Baptista et al., 2018; Duan & Xu, 2021; Frank et al., 2019; Shi-Nash & Hardoon, 2017)	*Explanation:* Predictive and prescriptive analysis seeks to identify future patterns and anomalies, based on CPS approaches and advanced data analytics. The objective is to improve efficiency and address customer demands by improving reactions to unpredictable events (Baptista et al., 2018; Shi-Nash & Hardoon, 2017). By combining domain knowledge with data-based approaches (e.g. machine learning models), the aim is to foresee trends, behavioural patterns, and correlations to predict critical events (Duan & Xu, 2021; Frank et al., 2019). Important decisions and actions are then taken (i.e. prescriptive knowledge) to avoid downtimes or minimise the personal costs of the respective services (e.g. number of contacts needed). *Example(s):* Recently, predictive maintenance services for production robots grew in importance. Especially during the COVID-19-pandemic, access to technical service experts was limited or prohibited at many organisations (I8).
Self-optimising products & production systems	A company’s ability to develop self-optimising products and cyber-physical infrastructures, which can make and deploy automated decisions (Kang et al., 2016; Shi-Nash & Hardoon, 2017; Vaidya et al., 2018; Waschull et al., 2020)	*Explanation:* This capability is about converting traditional machines to self-aware and self-learning machines to improve their overall performance and maintenance management (Vaidya et al., 2018). This builds on most of the technical capabilities described above. For automated production infrastructures, production involves several centralised or decentralised workstations that independently make decisions, dynamically allocate tasks, and seamlessly negotiate appropriate reactions to overcome problems. This also includes a degree of freedom in decision-making and the ability to learn from events or previously made decisions (Waschull et al., 2020). Most of the interviewed industry experts confirmed that they have not yet developed corresponding capabilities. *Example(s):* The use of autonomous and collaborative robots to automate handling, welding, and painting activities has already begun on several production sites (Wagire et al., 2020), as well as the implementation of self-managing smart factories (e.g. Bellini et al., 2021).
Autonomous provision of products & services	A company’s ability to automate the provision of products and services, including pricing mechanisms, proactive product-triggered communication, and real-time adaptation to exogenous events (Oztemel & Gursev, 2020; Shihundla et al., 2019)	*Explanation:* From a technical point of view, this capability is more complex than those discussed above, because it is derived from (almost) all of those capabilities. Interviewees often related it to concepts such as ‘smart factory’ or ‘dark factory’ because it establishes automatic solutions that will execute versatile operations. They will do so independently from location and provide the ability to react context-specifically to fast-changing customer needs (Lasi et al., 2014; Oztemel & Gursev, 2020; Shihundla et al., 2019). *Example(s):* When it comes to the autonomous provision of products and services, smart factories are known to implement this capability already. Manufacturing environments are equipped with automated systems to the extent that they do not require the presence of humans for anything more complex than simple tasks like removing parts (Oztemel & Gursev, 2020).

**Table 2:** Overview of support IS capabilities for Industry 4.0

Support IS capabilities for Industry 4.0		
Capability	Definition	Explanation & Example(s)
Strategy		
Strategic evaluation of customer & technology trends	A company’s ability to identify and assess digital customer and technology trends according to its business strategy (Neirotti et al., 2018; Schroeder et al., 2019)	*Explanation:* To ensure long-term competitiveness, a company must identify important trends in technology and customer demands. The key is to access different sources inside the company (e.g. technical and sales experts) and outside of it (e.g. major IT vendors). When assessing digital technologies, companies must systematically evaluate the values that such technologies or technological trends have for their business and customers (Schroeder et al., 2019). Furthermore, they must assess how customer needs and behaviours are changing due to new technological developments. For such evaluations, the potential use, technical feasibility, and related investments are all of importance. *Example(s):* According to our interviewed experts, identifying trends is often done by a business development unit that serves as a kind of radar for technology and customer trends. This capability could also be outsourced to experts in the respective field or research institutions *(I1, I3, I4).*
Data-based product & service innovation	A company’s ability to use customer and product lifecycle data to innovate products and services in a structured innovation process (Neirotti et al., 2018; Weking et al., 2020)	*Explanation:* Due to the emergence of digital technologies and the associated benefits of closer customer interaction and access to product lifecycle data, manufacturers can now leverage data to anticipate customer needs (Weking et al., 2020). They can, therefore, expand or refine existing product portfolios, provide value-added services, or seek out new co-creation opportunities (Neirotti et al., 2018). According to all of our interviewed experts, this translates into shorter time-to-market and higher innovation rates for products as well as services. *Example(s):* Two interviewed experts mentioned that their companies automatically monitor the usage of their products, using, for instance, corresponding vibration patterns to offer predictive maintenance solutions (I2, I4).
Readjustment of sales & service provisioning structures	A company’s ability to establish and adapt (organisational) structures to provide integrated product and service solutions	*Explanation:* This capability was emphasised and added by the interviewed experts because they identified it as one of the critical capabilities for succeeding in marketing and providing new digital product and service solutions, especially when the service is not a by-product anymore. The experts stressed the (organisational) readjustment because most manufacturers of the service and sales department are still focused on the (one-time) sales of products instead of providing continuous service relationships. Although this aspect has not been extensively researched so far, some work points out that the reorganisation behind and automation of service offerings is crucial to ensure service availability to the customer and profitability to the manufacturer (e.g. Cimini et al. (2020). *Example (s*I1 and I4 pointed out that marketing digital service solutions differ from marketing physical products, requiring the adaption of new approaches such as live demonstrations. I8 added that the company needs to adapt roles (e.g. for sales representatives) and systems (e.g. incentives) accounting for digital services’ success.
Strategic leveraging of partner networks	A company’s ability to rethink, use, and adjust its business network to increase internal efficiency and extend its value proposition (Wagire et al., 2020; Zacca et al., 2015)	*Explanation:* To go with the times, manufacturers have to learn how to initiate, develop, and use new business partnerships, such as a portfolio of third-party technology providers that enable them to complement their own capabilities (Zacca et al., 2015). As a result, manufacturers become part of digital ecosystems that integrate the resources of various partners to ensure that additional capabilities are available when co-creating Industry 4.0 solutions (Cainelli et al., 2012; Wagire et al., 2020). *Example(s):* Our interviewed experts made the point that some key questions have to be asked when deciding whether to insource or outsource IS capabilities. These questions concern not only the ways in which manufacturers can differentiate themselves from competitors, but also the access to experts on whichever capability may be required, and of course, the issue of long-term investments and who may provide these (I4, I7, I10).
Technology		
Seamless operations & process digitalisation	A company’s ability to ensure operational efficiency and to continually digitalise processes (Schroeder et al., 2019; Wagire et al., 2020; Waschull et al., 2020)	*Explanation:* Digital technologies make it possible to improve operational efficiency in the organisation, and indeed in the process of dealing with customers (I1, I4). This capability requires employees to understand, monitor, and synchronise integrated processes (Schroeder et al., 2019; Waschull et al., 2020). Our interviewed experts emphasised the challenge of digitalising support processes connected to new product and service solutions, given that new digital offerings also afford different processes and delivery systems. *Example(s):* By better connecting available logistics data, the established rationale of planning supply by first looking at demand can be replaced with a data-based solution that takes over the scheduling process (Waschull et al., 2020). Specific implementation examples include the automation of a customer support process for contract cancellations (I1) and the automation of billing processes (I4). Both were previously performed manually.
Seamless human-machine collaboration	A company’s ability to provide customised and flexible user interfaces as well as enable seamless collaboration between humans and machines (Patterson, 2017; Schuh et al., 2020; Wagire et al., 2020; Wittenberg, 2016)	*Explanation:* User-friendly solutions for employees and customers alike should be implemented for an intuitive and seamless collaboration between humans and machines (Schuh et al., 2020; Wittenberg, 2016). The design of related interfaces and systems needs to consider psychological and cognitive human requirements (Patterson, 2017) to ensure it eases human-to-machine communication in various roles (Wagire et al., 2020). Our industry experts highlighted the importance of this because easy-to-use interfaces and systems relax the requirements of the experts handling the machines (I2, I3). *Example(s):* User-friendly interfaces, such as rotary controls and instructional displays, are an upcoming topic of notable significance to the further development of the manufacturing system (I4, I8).
Governance of data, product security & liability	A company’s ability to prepare, prevent, recover, and learn from actual or potential cyber security threats (Babiceanu & Seker, 2016; Liu et al., 2020; Schuh et al., 2020; Weber et al., 2019).	*Explanation:* Due to the implementation of connected products and the associated data exchange within and across organisational boundaries, the protection of intellectual property plays an essential role, as does the protection of customer and employee data from threats such as cyber-attacks, unauthorised access, and industrial espionage (Liu et al., 2020; Schuh et al., 2020). From an ex-ante perspective, the ‘prepare and prevent’ strategy provides a comprehensive analysis of the present threat situation and the implementation of security measures that cover product administration and data ownership structures as user authentication mechanisms. As for ex-post countermeasures, the ‘recover and learn’ strategy makes it possible to mitigate the harm caused by such attacks (Babiceanu & Seker, 2016). *Example(s):* This capability can include encryption, authentication, and authorisation measures that help establish secure communication protocols (Wagire et al., 2020). The company of I3 decided that this does not necessarily mean that the implementation of cyber-security measures must be done internally, but rather that the governance mechanisms which guarantee maintained control are essential to its organisation.
Intra-organisational information exchange	A company’s ability to achieve a seamless and context-driven exchange of relevant information (Emmanouilidis et al., 2019; Endert et al., 2014).	*Explanation:* An efficient and user-oriented information exchange is necessary to make innovative product and process improvements. Only relevant data should be made available to employees to increase data consumption efficiency and better manage the increasing amount and complexity of data (Emmanouilidis et al., 2019). *Example(s):* One possible solution is visually enhanced data, such as dashboards or task-specific user interfaces which deliver the appropriate amount of information for a specific context (Emmanouilidis et al., 2019). Our interviewed experts suggested that this could be implemented by displaying only the information necessary to guide a service technician through a repair process (I2) or relevant key-performance indicators on a screen above production units (I4).
Human Resource		
Established mutual trust, adaptability, & openness	A company’s ability to establish, maintain and promote mutual trust within their organisation as well as with their customers in order to adapt to new developments and trends (Bienhaus & Haddud, 2018; Weber et al., 2019)	*Explanation:* An essential building block of Industry 4.0 is the mutual trust established among all involved parties, including employees, customers, and ecosystem partners (Bienhaus & Haddud, 2018). Industry 4.0 refers to highly interconnected supply chain ecosystems in which trust is an essential factor due to the high incident rate of interactions and data sharing (Weber et al., 2019). Such trust is critical in customer relations because smart products not only require the willingness to share data (e.g. usage data). They also provide the foundation for further exchange in new types of product and service offerings. *Example(s):* As one industry expert highlighted, the value of a feature needs to be visible and made transparently. Otherwise, it will not be easy to convince the customer for trying something new or sharing sensitive data (I3).
Leadership for change & agility	A company’s ability to find the right form of leadership in a complex and fast-paced business environment facilitates an agile working context in which to adapt well to change (Benešová & Tupa, 2017; Shamim et al., 2016; Wagire et al., 2020)	*Explanation:* Companies must employ an agile leadership that enables their employees to accept and embrace change (Wagire et al., 2020). Both the literature and our interviews provide evidence that the social attitudes of adaptability and openness need to be fostered. As Industry 4.0 continues its complex and unpredictable progress, open management must adapt to new technological developments (Shamim et al., 2016). Furthermore, managers and team-leaders should encourage openness towards continuous learning and interdisciplinary teamwork (Benešová & Tupa, 2017). *Example(s)*: Two industry experts mentioned challenges they currently face in establishing a management culture supported by all and minimises the fear of employees losing their jobs to technology (I1, I2).
Profound digital literacy of employees	A company’s ability to improve its employees’ knowledge of digital technologies and their competence in evaluating and implementing them (Bonekamp & Sure, 2015; Prifti et al., 2017; Wagire et al., 2020)	*Explanation:* Since working in Industry 4.0 increasingly requires digital literacy, organisational structures that facilitate the development of both general and role-specific digital competencies must be established (Bonekamp & Sure, 2015). Digital literacy, however, refers not only to comprehensive technological knowledge but also to the ability to solve complex technological problems, learn new methods, and apply them (Prifti et al., 2017). *Example(s):* This involves knowledge of the right choice of collaboration tools (I9) and knowledge of technological possibilities (I10), as well as the secure handling of digital technologies when dealing with sensitive data (I8).
Fostering interdisciplinary teamwork	A company’s ability to empower employees to communicate and collaborate in interdisciplinary setups and to involve different roles in innovation processes (Waschull et al., 2020; Weber et al., 2019; Xu, 2020)	*Explanation:* Due to the interdisciplinary nature of Industry 4.0 projects, there is a need for collaboration between various roles and departments, be it within a company or beyond. This is why it is important for manufacturing companies to promote an interdisciplinary approach for creating and sharing knowledge. This can be achieved by implementing open innovation processes involving experts from various domains (Waschull et al., 2020; Weber et al., 2019; Xu, 2020). *Example(s)*: The breaking up of silo structures, the establishing of cross-organisational setups for idea crowdsourcing (I3), and the provision of tools for virtual collaboration among people in a variety of locations all became a necessity in the lockdown periods during the COVID-19-pandemic (I8).

## Demonstration

We applied the IS capability framework with a German manufacturer for sheet metal and tube processing solutions, to be called German Manufacturer for anonymity reasons, or GM for short. We did this to demonstrate the framework’s practical use, which is to support manufacturers in their Industry 4.0 transformation efforts. Specifically, GM’s Chief Transformation Officer (CTO) used the framework to analyse the status quo and desired target state of GM’s Industry 4.0 capability development and reflect on the various chances and challenges associated with their existing and planned products and services projects and initiatives. By assisting in this process, we aimed to evaluate the framework’s understandability and applicability (Sonnenberg & vom Brocke, 2012).

### Background Information on GM

GM is a globally leading manufacturer for industrial machines, headquartered in Germany. The company is well-established, has over 10,000 employees on its books, and operates globally through more than 70 subsidiaries. The larger area of GM’s product portfolio comprises sheet metal and tube processing solutions which involve machines for bending, punching, and laser processing. GM started its Industry 4.0 transformation more than a decade ago and has already provided a significant number of integrated digital services to complement its products and extend its value proposition. To ensure optimal strategy and organisational development, however, the CTO had to assess and analyse the progress of its Industry 4.0 transformation. Through our capability assessment, the CTO intended to ensure that all of the company’s ongoing and planned capability developments were aligned with its goal to defend its role as a technology leader in the market.

### Capability Assessment

The capability assessment was conducted two-step and in close collaboration between the CTO and the author team. This involved several interviews and workshops between September and December 2020. All discussions were conducted via video conference, the recordings of which were analysed and the most important insights documented. The latter were subsequently discussed and consolidated with the CTO (see Table 3). The two steps were as follows:

- Step 1 - Capability assessment of status quo and desired target state:

**Table 3:** Framework application at GM

Capability	Level of maturity					Products, services & initiatives	Status quo evaluation of capabilities
	1	2	3	4	5		
Primary IS capabilities for Industry 4.0							
Connect & Store							
Product connectivity & system inter-operability				**x**	**x**	Remote support services	**Chances:** GM’s customer’s (i.e. discrete manufacturing machines) can be equipped with solutions for remote management (connectivity); solutions provide interoperability with major MES/CAD/CAM systems **Challenges:** Regulations and customer security policy often limit access to products; it can prove challenging when various connectivity platforms need to be run in parallel
Seamless connection to customers			**x**	**x**		Webshop; Smart service apps	**Chances:** The webshop can be used to order consumables and spare parts, which is a high-profit business; the company can free up the workforce by automating such tasks as technical support services or the documentation of maintenance actions, all of which can be done with the smart service app **Challenges:** Getting customers to use digital channels involves change for small and medium businesses and their customer relation departments, which have spent decades performing the same activities via phone and fax
Continuous collection & storage of real-time data			**x**	**x**		Track & trace services	**Chances:** Scalable solutions for data storage are in place and can be adapted to the specific needs of the respective customer and industry; learning from customer behaviour on the broader industry (e.g. regarding frequency and access) can help a company find the right solution with greater ease and speed **Challenges:** The key is to generate value from data (e.g. digital services) and then monetise that value because the upfront costs for data storage can be immense (e.g. sensor data); however, there are still major hurdles that can get in the way of convincing customers to pay for complementary services
Understand & Act							
Automated processing of (un-)structured data		**x**	**x**			N/A	**Chances:** The evermore established standards for interfaces (e.g. OPC UA) on the shopfloor can simplify the access to different data sources as well as their aggregation **Challenges**: It is still challenging to aggregate data from different machines, which is due in part to the varying ages and constructions of the machines and in part to the limited compatibility of multiple manufacturers and software providers
Descriptive & diagnostic analysis		**x**	**x**			Performance & condition monitoring apps	**Chances:** Performance indicators that make it possible to measure a product’s condition or quality are defined upon consideration of customer feedback **Challenges:** Even when one’s own dashboard is in place, it is more important to integrate it into the customer’s solutions, which is not always easy
Automated monitoring & reasoning		**x**	**x**			Material analytics app	**Chances:** First analytical solutions are in place to optimise production processes and achieve direct savings for the customers (e.g. mapping of materials to product orders; optimising the use of the material) **Challenges:** Past a certain degree of complexity, automation requires a lot of customer- and process-specific adaption
Predict & Self-optimise							
Predictive & prescriptive analysis	**x**		**x**			Predictive services based acoustic data	**Chances:** Based on sensory data (e.g. acoustic data), a problem occurring in the cutting process can be detected, after that the likelihood of machine failure can be predicted – acoustic anomalies detection promises to have a significant potential **Challenges:** This capability is still an investment topic for GM, and the CTO is unsure whether customers will pay for it – in any case, it will require a lot of patience to see a return on investment
Self-optimising products & production systems	**x**		**x**			AI-specific services	**Chances:** AI-based services help reduce costs and minimise unpleasant tasks currently performed by staff in order to defend GM’s role as a market leader **Challenges:** The requirements for data quantity and quality still pose great challenges to the company; collaboration with research institutes and other partners is required to use the full potential of this capability
Autonomous provision of products & services	**x**		**x**			Smart factories	**Chances:** The company can use smart factories to demonstrate new product solutions; these can take over during production peaks or produce small batches for specific customer needs (e.g. prototypes) **Challenges:** It is not yet clear whether the high investments will pay off; it can also be challenging to justify such high investments to the supervisory board; the degree to which “dark factory” approaches (i.e. without any human interventions) are desirable is unclear
Support IS capabilities for Industry 4.0							
Strategy							
Evaluation of customer & technology trends			**x**	**x**		GM’s trend radar	**Chances:** The company can use its innovation radar based on customer surveys and market research to identify and prioritise trends; using personas for different customer segments helps to improve customer understanding **Challenges:** It may prove difficult to ensure that research is not driven too much from the inside (i.e. domain experience) as the lack of an external perspective may make it challenging to recognise disruptive trends
Data-based product & service innovation		**x**		**x**		Inter-disciplinary business development team	**Chances:** An interdisciplinary business model team could be appointed to bring together the necessary skills to develop data-based business models (e.g. adapt digital innovation methods or new business models) **Challenges:** Breaking the mould when developing new products challenges existing structures and people’s understanding of their roles
Readjustment of sales & service provisioning structures		**x**		**x**		N/A	**Chances:** End-to-end services can be introduced for new product and service solutions **Challenges:** It may be challenging to establish new service structures, especially when unique expertise has to be developed and the staff increased; further challenges may arise as old and newly developed systems need to be serviced simultaneously
Strategic leveraging of partner network	**x** **x**					Pilot projects for new business models	**Chances:** Pilot projects can collect first experiences and insights, for example, with an important customer and globally acting financial service provider, which can help to pioneer a new business model (i.e. pay-per-part model) **Challenges:** There may be some difficulties in finding the right service/implementation partners, but less so with the technology partners; it may also prove challenging to define a clear technology target and align all internal stakeholders accordingly
Technology							
Seamless operations & process digitalisation			**x** **x**			Focus on ordering processes	**Chances:** Management has identified the great potential for cost savings with automation of support processes before and after manufacturing; faster and more efficient order and billing processes can also enhance customer experience **Challenges:** Speeding up such processes can be hampered by silo structures
Seamless human-machine collaboration			**x**	**x**		Tests with camera systems	**Chances:** Novel camera systems have entered the test phase and already proven capable of perceiving their surroundings in ways similar to the human eye in that they can recognise depth, motion, and surface structures, which makes them valuable to machine operators as it lets them see more clearly which tasks to prioritise **Challenges**: Finding experts for computer vision and related domains may be difficult and costly
Governance of data, product security & liability			**x**	**x**		Vulnerability management	**Chances:** As security can make the decisive difference for customers, it is a selling point when vulnerability management is in place to process any vulnerabilities and security gaps in one’s own or third-party components **Challenges:** Data usage agreements are a challenging topic since customers raise concerns about liability issues
Intra-organisational information exchange			**x** **x**			Internal fairs and exchanges	**Chances:** Conducting internal exchanges among digital project managers were very helpful in managing interfaces **Challenges:** Company communication platforms and channels do not yet support the desired exchange
Human Ressource							
Established mutual trust, adaptability, & openness		**x** **x**				E-learning platforms	**Chances:** By installing e-learning platforms for off-line and online learning in multiple languages, content can be offered 24/7; content can then be made accessible for other countries and languages with reasonably low effort **Challenges:** First, one has to win customers’ trust in business models that provide new ways of delivering value
Leadership for change and agility		**x**	**x**			Trainings and academies	**Chances:** New skills can be developed in an academy format structured around digital technologies **Challenges:** It may prove challenging to train seasoned managers in agile methods; before new approaches are adopted, one has to invest time and practice to be adopted
Profound digital literacy & digital competences			**x** **x**			Trainings and academies	**Chances:** Optimally equipped seminar rooms; training on the machine directly **Challenge:** Bringing an organisation on a basic skill level is a ‘mammoth task’ and affords high investments
Fostering interdisciplinary teamwork			**x**	**x**		Inter-disciplinary institutes	**Chances:** Interdisciplinary institutes that bring together different faculties can stimulate people to think ‘outside the box’; the pathways of collaboration can be notably shortened (e.g. shared cafeteria, conference rooms) **Challenge:** Bringing experts from different domains together and getting them to produce innovative work can require more than shared rooms

**x** = Status quo (today); **x =** target state (in 3 years); N/A = no information available

The framework was extended to generic maturity levels to assess each capability’s status quo and desired target state in 3 years. To facilitate simple implementation, a five-point Likert scale was used for the varying levels of maturity, a methodology the success of which has already been proven by the likes of Wagire et al. (2020) and Schumacher et al. (2016). On this scale, ‘1’ stands for ‘No or low level of maturity – being a novice in this topic’, whereas ‘5’ represents ‘a high level of maturity – being an expert in this topic’.

- Step 2 - Reflection on ongoing projects, chances, and challenges:

The CTO used the IS capability framework to map existing products and services and ongoing projects and initiatives to each capability dimension to reflect on how well the capability development for Industry 4.0 is currently being operationalised. Next, the CTO and the authors reflected on which chances and challenges arise with the respective Industry 4.0 capabilities.

The assessment (Table 3) showed that GM’s range of Industry 4.0 products, services, projects, and initiatives is already relatively rich. The CTO confirmed that the framework was useful in making this assessment and taking a holistic view of these multiple aspects, mainly because he found it easy to understand and apply. According to the CTO, the distinction between primary and support capabilities ‘is a well-established view in industry and familiar among managers’. This supports common understanding. When discussing the framework’s understandability, the CTO confirmed that the short explanations were helpful. However, their terminology might have to be refined in the application process to account for company-specific terms.

## Discussion

Industry 4.0 is recognised as one of the most significant paradigm shifts in the manufacturing domain and associated with the potential to disrupt entire value networks. Yet, manufacturers still struggle with realising its full potential. Although the literature on Industry 4.0 can be considered mature and the ‘why’ and ‘what’ appear to be straightforward, the ‘how’ is still evolving and depends on the build-up of required IS capabilities. Although there is substantial work and knowledge on Industry 4.0 (e.g., Li (2018), Xu et al. (2018), Weking et al. (2020)), an IS perspective on Industry 4.0 has so far been insufficiently researched. This also explains why companies, whose strengths lie in manufacturing-related core capabilities, face difficulties understanding which IS capabilities are needed to realise their transformation strategies towards Industry 4.0.

To advance the understanding of Industry 4.0, we developed IS capability framework. We followed an explorative approach to identify relevant IS capabilities in literature according to the well-established approach of Wolfswinkel et al. (2013). The resulting framework was iteratively refined and evaluated with senior experts from eight German manufacturers differing in size and industry. Two were eminent academics who specialised in application-oriented research on Industry 4.0. To demonstrate the practical use of this IS capability framework, it was applied in the context of a capability assessment with the CTO of a German manufacturer dealing in metal and tube processing solutions. The most significant implications that became apparent during the development, evaluation, and demonstration of the framework are outlined in the following pages.

### Theoretical Implications

The core theoretical implications of our study are twofold as they complement existing knowledge on Industry 4.0 capabilities and lay the foundation for further theorising:

#### Complementing Knowledge on Industry 4.0 by Providing a Holistic Overview of Relevant IS Capabilities:

The literature on Industry 4.0 can be deemed mature as it offers relevant conceptual work (i.e. frameworks, maturity models) that describe and structure the complex affordances of Industry 4.0. What it does not provide, however, is a comprehensive theoretical overview of the IS capabilities required for Industry 4.0, although this is somewhat overdue, given the diverse nature of the topic and the required collaboration across long-established domain boundaries (Ghobakhloo, 2018; Lund & Karlsen, 2020). To look beyond the familiar practice- and application-oriented perspectives, we focused on making a pivotal contribution to the literature: a holistic IS capability framework based on a thorough and structured literature review that would enable us to summarise the current state of research and build on its findings. Furthermore, by structuring capabilities into primary and support capabilities, we set out to complement the often rather technical focus on capabilities that are taken when dealing with digital technologies and products (i.e. primary capabilities). We did this in the hope that a clear eye for the practical use of such a framework would help establish the holistic perspective required by manufacturing companies.

In the following, we briefly describe how we used and advanced the existing knowledge on required IS capabilities for Industry 4.0. To develop our framework, we identified the relevant IS capabilities in publications with specific focal points, such as the information management capability framework of Stich et al. (2017) or the Industry 4.0 competency model of Prifti et al. (2017). We took their work a step further by first breaking down their rather general capabilities and then describing them in more detail. For example, the capability ‘data analysis’ in the framework of Stich et al. (2017) is broken down into several different ones, i.e. ‘descriptive & diagnostic analysis*’, ‘*automated monitoring & reasoning*’, ‘*predictive & prescriptive analysis*’*. Furthermore, by taking a holistic perspective we look beyond their rather narrow focus on technical capabilities (Stich et al., 2017) or employee competencies (Prifti et al., 2017). In other areas, we looked at work predicated on a progress-oriented perspective, such as the maturity models of Schumacher et al. (2016), Wagire et al. (2020), and Schuh et al. (2020). These provided us with valuable input for this study since they all take a more holistic perspective that appreciates technological and cultural aspects and accounts for IS capabilities in this context. To name but a few examples, our support capabilities concerning digital literacy and strategic partnerships were influenced by the capabilities that Wagire et al. (2020) identified in relation to people and culture (e.g. ‘digital skill and qualification’) and organisational strategy (e.g. ‘collaboration’). Beyond that, the IS capabilities which Schuh et al. (2020) identified as being part of four major Industry 4.0 pillars helped us clarify our thoughts on numerous primary capabilities, such as ‘product connectivity & system interoperability’, ‘continuous collection & storage of real-time data’, and ‘automated processing of (un-)structured data’. As we found them wanting in several key areas, however, we added certain new IS capabilities like ‘autonomous provision of products & services’, which we developed after conducting a thorough literature review. A final observation worth making here is that, while most new work is either deducted from the broader literature or produced in consultation with expert panels, we combined the advantages of both approaches. Thereby, we combined our theory-based knowledge on Industry 4.0 capabilities with expert insights on hitherto unconsidered IS capabilities, such as the ‘seamless connection to customers’ and the ‘identification of customer and technology trends’. By harnessing our discourse to the real-world examples our interviewed industry experts provided we ensured a close connection between theoretical and application-oriented research (Corley & Gioia, 2011; Moeini et al., 2019).

#### Laying the Foundation for Further Theorising:

As mentioned above, literature on Industry 4.0 already provides relevant conceptual work (i.e. frameworks, maturity models) that describe and structure the complex affordances of Industry 4.0. The developed capability framework for Industry 4.0 adds to existing work as it lays the ground for further theorising and understanding Industry 4.0 transformations. Further, it builds the foundation for deriving specific capability design recommendations and thus represents an additional critical piece of the puzzle to implement Industry 4.0 transformations in an even more structured and targeted manner (Gregor, 2006). The following table gives an overview of the framework’s full contributions and indicates how it can be used as a foundation for further research Table 4:

**Table 4:** Overview of the IS capability framework’s stimuli for further research

IS capability framework’s contribution	Stimuli for further research	Explanation
The modular structure of capability areas and dimensions	Framework extension	Fellow researchers should extend the framework by adding relevant capabilities
Provision of IS capabilities derived from domain literature and evaluated by senior industry executives	Maturity model extension	Fellow researchers should extend existing maturity models for Industry 4.0 by taking hitherto unconsidered IS capabilities into account. We developed the framework based on a structured literature review and expert interviews with senior executives as outlined above. The overview of relevant capabilities from all relevant work and the supplementation of important capabilities by the industry experts supports fellow researchers in extending (general) framework and maturity models from Industry 4.0 transformations. Alternatively, an IS-specific maturity model for manufacturers could also be developed.
Provision of IS capabilities that senior industry executives have evaluated regarding relevance, applicability, and usefulness	Development of measurement approaches	Fellow researchers should develop measurement instruments to support practitioners in assessing the progress in capability development.
The framework and its demonstration at GM provide first insights into its usefulness along with lessons learned by a significant industry player during its Industry 4.0 transformation.	Conducting case studies	Fellow researchers should use the Industry 4.0 capability framework for rigorously conducted case studies to provide insights on the chances and challenges in Industry 4.0 transformations by focusing on building up required IS capabilities. Further, researchers should identify and carve out general success factors in the Industry 4.0 transformation.

### Practical Implications

The evaluation and demonstration of the framework have shown it to have three major practical implications:

#### Managers could use the IS Capability Framework to develop their Industry 4.0 Capability vision and compare it to the status quo:

Our framework enables managers to develop their Industry 4.0 capability vision systematically. As the application at GM has indicated, managers can use the framework to assess their company’s status quo as well as the desired target state of each capability. As one of our interviewed experts stated: ‘Knowing exactly where you are and want to be in a few years […] is the input needed when developing or adapting the digital strategy […], since currently when it comes to the implementation of digital technologies, in our organisation a late-mover approach is being taken’ (I1). With the help of our framework, manufacturers can assess the status quo of each capability, be that with regard to subsidiary elements of their organisation or its entirety. Not only does this shed light on the progress of the organisation’s transformation. It also supports management in ensuring it doesn’t neglect relevant capabilities. Furthermore, it lays the foundation for defining suitable process and progress indicators, which should prove helpful in navigating through the often complex Industry 4.0 transformation, a journey on which many manufacturers are still coming up against significant hurdles.

#### Managers could use the IS Capability Framework to decide on in-house versus partner capability development efforts:

To reach their Industry 4.0 capability targets, managers need to decide which IS capabilities to develop internally and which to access through external partners. Since the resources and skills required for internal capability development are often limited, and since external digital service providers with relevant expertise often possess competitive advantages in their digital capabilities, a thorough assessment should be given a high priority. On that note, industry expert I8 stated that his company ‘got a bloody nose […] because, in the beginning, we thought we had to do everything ourselves as we were used to’. Not only does our IS capability framework provide the holistic perspective required to perform a strategic assessment and avoid further bloody noses. It also supports managers in considering all relevant capabilities. As expert I10 mentioned, it provides ‘a good structure for a first glance assessment of external partners by using the framework for the analysis of their offered products and services […] and a better understanding how the offering complements our capabilities’.

#### Managers could use the IS Capability Framework to assess their Industry 4.0 IS Capability development regularly:

Since Industry 4.0 transformations take time, the systematic and regular assessment of its progress is of great importance. As the framework’s application with GM has already indicated, identifying existing and planned products, services, projects, and initiatives, when mapped to the respective capability dimensions, provides managers with a holistic overview of how current trends and ambitions factor into the company’s Industry 4.0 strategy. What is more, this facilitates a simple fit-gap-analysis, which provides valuable awareness of the company’s present strengths and weaknesses, and indeed of how their planned products, services, projects, and initiatives might best be adjusted. A final point worth making in this context is that our interviewed industry experts also thought of using such insights to adjust incentives for Industry 4.0 projects (I10) and management reports (I2).

## Limitations and Further Research

Although we have endeavoured to be as thorough as possible in our research, this study has inevitable limitations the recognition of which shall point our peers in the direction of further beneficial research. The first of these limitations lies in the restricted nature of our interview circle. Future research could expand this circle by including more experts from academia and industry. If, for instance, industry experts from other countries were consulted, their varied experience of different industry structures would broaden the appeal and effectiveness of our framework, either by confirming the IS capabilities we have included or by adding others. A further limitation of our study concerns the type of artefact (i.e. framework) used to structure and describe the relevant IS capabilities for Industry 4.0. Being a framework, it provides a descriptive overview of the relevant capabilities but does not offer specific guidance on achieving or developing them. Further research would, then, do well to focus on prescribing advice on selecting, evaluating, and completing the IS capabilities for Industry 4.0. A third limitation of this study is its explorative nature since we consulted industry experts to identify capabilities that have not yet been covered in the literature. We conclude that these capabilities provide excellent opportunities for future research to deepen the understanding of IS capabilities, which is essential to making the most of Industry 4.0. The fourth and final limitation that future researchers may wish to consider is that we have taken a static view of Industry 4.0 capabilities without accounting for interdependencies in terms of time or content. Therefore, future research could investigate those interdependencies of various IS capabilities and their causal relationships. For example, we consider capabilities like ‘product connectivity and system interoperability’ foundational capabilities and prerequisites to enabling more advanced capabilities, such as ‘self-optimising products & production systems’. Shedding light on these dependencies would also support practitioners in prioritising the development of different Industry 4.0 capabilities.

These limitations notwithstanding, we are confident that the framework developed in this study is an essential measure in structuring IS capabilities for Industry 4.0. We hope that it will support manufacturers in their Industry 4.0 transformation and assist fellow researchers as they continue their work at the interface of Industry 4.0 and IS capabilities.
